# A Longitudinal Observation of the Influence of Michigan Sports Concussion Law on Parents’ Knowledge and Perception of Sport-Related Concussion

**DOI:** 10.51894/001c.22067

**Published:** 2021-04-13

**Authors:** Virginia LaBond, Karyn Liebsch, Brian West, Dane Caputo, Kimberly Barber

**Affiliations:** 1 Emergency Department Ascension Genesys; 2 Emergency Medicine Ascension Borgess; 3 Emergency Medicine Ascension Genesys; 4 Emergency Medicine Banner Thunderbird Medical Center; 5 Clinical and Academic Research Ascension Genesys

**Keywords:** parental attitudes, traumatic brain injury, athletics, youth, sports, concussion

## Abstract

**INTRODUCTION:**

In 2013, Michigan enacted legislation requiring parents and athletes to receive educational material concerning sport-related concussion (SRC). The aim of this study was to examine trends in concussion knowledge of parents from one community following implementation of Michigan’s Sports Concussion Laws (MSCL).

**METHODS:**

A convenience sample of parents of students from a suburban school district were surveyed via district email at one year and five years post MSCL implementation. Results were compared to an equivalent 33-item survey obtained prior to the law. Individual questions were compared between the three surveys using Chi-square analysis with statistical significance observed at p < 0.05.

**RESULTS:**

A total of 381 responses were obtained from the one-year post-MSCL (1yMSCL) and 178 in the five-year post-MSCL (5yMSCL) survey. Awareness of district policy regarding concussion was significantly higher after implementation of the MSCL (i.e., 77% at 1yMSCL and 71% at 5yMSCL) compared to prior 18% pre-MSCL (p < 0.0001). Respondents to the 5yMSCL survey were also significantly more aware of medical guidelines surrounding “return to play” after SRC compared to 1yMSCL (84.8% v 78.7%, p = 0.01). At 5yMSCL, significantly more respondents agreed that head injuries could cause more brain damage to children than adults (86.5% v 78.7% at 1yMSCL, p = 0.03). Finally, most parents at both survey periods rated the concussion educational material as the most helpful information source regarding SRC.

**CONCLUSIONS:**

Based on these results, parental knowledge awareness appears to have increased concerning awareness of medical guidelines for SRC and potential brain damage risks to children after enactment of the MSCL.

## INTRODUCTION

In 2016, the National Football League (NFL) settled for almost 1 billion dollars over thousands of lawsuits regarding mild traumatic brain injury (mTBI), better known as “concussion.” At the time of this paper, these settlements are still ongoing. These lawsuits began after growing awareness of the long-term consequences of concussions (e g., chronic traumatic encephalopathy (CTE). For example, a 2017 study showed that 110 of 111 former NFL players had CTE evident on brain autopsy.^1^ This finding helped increase mainstream media attention on concussions during contact sports for adults, adolescents and children.

Organized sports have long been an integral element of American culture for children and adolescents. Although football has gained the most attention for highest incidence of concussion, many other sports (e g., soccer, lacrosse, baseball, softball, ice hockey, and others) have been shown to impose significant concussion risks.^2–5^

For over two decades, there have been guidelines and sideline assessment tools (e g., Sports Concussion Assessment Tool, 5^th^ edition (SCAT5) available to coaches, sports trainers and physicians to examine athletes after head trauma.^6–9^ It has only been recently that emphasis has been placed on training school professionals and parents who may have more continued contact with children.^10,11^ For example, the 2003 CDC *HEADS UP* program to educate health care professionals has rapidly expanded to school-wide programs and partnerships with large sports organizations.^12,13^ In 2011, however, a study completed by the first author of this paper demonstrated that survey respondents in one community still had limited knowledge regarding concussions.^14^

Since that study, Michigan and several other states have passed legislation to improve parental education of sports-related concussion risks in youth athletes.^15^ Michigan Public Acts 342 and 343 were enacted in 2013 and amended in 2017 with Public Act 137. The laws were entitled 2013 Sports Concussion Laws (MSCL).^15^ In this legislation, a youth athlete was defined as an individual who participates in an athletic activity and is under 18 years of age. Legislators required that all “organizing entities” (e g., schools, park and recreation departments, other groups organizing sporting events for youth athletes) provide an educational handout be distributed to parents of youth athletes and that both parents and children acknowledge reception of the handout before their child can participate in school-sponsored athletics.^16^

### Purpose of Study

Following implementation of the MSCL in 2013, the authors investigated parents’ knowledge and attitudes regarding sport-related concussion (SRC) at one-year post-MSCL (1yMSCL) and five years post-MSCL (5yMSCL). The aim of this descriptive correlational study was to evaluate relative changes and trends in parental knowledge and perceptions of SRC after maturation of the MSCL. The authors had hypothesized that parental knowledge levels regarding the MSCL would significantly increase from maturation of the MSCL and heightened media attention of SRC.

## METHODS

The authors compared the results of their serial one (i.e., 2014) and five-year (i.e., 2018) post-MSCL surveys with survey responses obtained from the authors’ previous 2011 study.^14^ The study described in this paper included these later surveys administered to parents and guardians of student athletes enrolled in the suburban, middle-class, Clarkston Michigan Community School District. The district had approximately 7100 enrolled students at seven elementary schools (K-5), two middle/junior high schools, and two high schools. The district also had a virtual academy and an Early Childhood Center serving children ages 3-5.^17^

Prior to dispersing surveys, project approval from the superintendent of the school district was provided. Institutional Review Board approval was obtained from the primary author’s health system. Individuals who were enrolled in the school district email list were sent a 33-item electronic survey link created by the authors.

Respondents were asked to follow a link to a SurveyMonkey™ website to complete the questionnaire (see Appendix).

The first informational item described the nature of the survey and asked for consent. The last question provided respondents the opportunity to select a school within the district to win fifty free bicycle helmets. This was offered as a small approved incentive for parents to complete the survey and the school with the most votes would receive the helmets.

At the time of the 2014 survey, there were approximately 6,500 emails within the school district list. The email system potentially included duplicate listings, non-active listings, or listings for more than one parent/guardian per student. The survey link was sent two to three times over several weeks to maximize responses.

Identical surveys were used for both 1yMSCL and 5yMSCL surveys to ensure comparability. All survey questions were the same as in the pre-MSCL survey with the exception of two additional items that asked respondents about their awareness of the MSCL and usefulness of various informational resources concerning SRC.

Data sets were collated and compared. Measures were indexed on dichotomous, three-point and four-point Likert Scales. Scores from individual questions and combined areas were compared between survey periods. A McNemar Test for paired proportions analytic procedure was used to compare pre-MSCL responses to current responses. Individual questions were compared between the three surveys using Chi-square analyses with statistical significance set at p value ≤ 0.05. The authors had generated a minimum estimated sample of 170 respondents during each survey time period to achieve power of 80% to detect whether there was a significant relative change in awareness of 35% for a total of at least 340 respondents across each survey subgroup.

## RESULTS

Approximately 6,500 email addresses were utilized using the school district electronic mailing list. The number of addresses that are duplicates, inactive and/or unattended is unknown. A total of 381 discrete survey responses (5.9% of the email distribution list) were obtained for the 1yMSCL survey, and 178 (2.7% of list) for the 5yMSCL survey. The pre-MSCL study had 245 respondents.

Of our 178 5yMSCL respondents, the vast majority were female (160 or 90%) with a mean age of 44 (SD 9.06). Although most respondents (i.e., 90%) identified themselves as parents or guardians, a small percentage were reportedly school employees or other family members. Over 80% (n=142) of total respondents had reportedly earned a bachelor’s degree or higher. The demographic characteristics of the 5yMSCL respondents were not significantly different from the 1yMSCL respondents. Of the 5yMSCL respondents, 30 (17%) reported personally having sustained a prior head injury and nearly one-third (31%) of their student-athletes reported such injury.

Overall, respondents had students in fairly similar distributions across preschool to high school categories (Table 1). The number of students exceeded the number of parent/guardian respondents due to having more than one child enrolled at the time of the survey. The most reported ’played' sports were soccer (47%), swimming (35%), ski/snowboarding (33%), basketball (33%), football (30%), and baseball (30%), followed by 26 other sports. (Table 2) This was similarly distributed between the 1yMSCL and 5yMSCL surveys.

**Table 1:** Student-athlete Grade Level

**Grade**	**Number of Athletes Number (% of total)**
**Preschool/kindergarten**	36 (12.7)
**Grades 1-5**	79 (27.8)
**Grades 6-7**	51 (18.0)
**Grades 8-9**	44 (15.5)
**Grades 10-12 (High School)**	74 (26.0)
	Total: 284*

*Number of students exceeds respondents due to multiple child households

**Table 2:** Sports Participation

**Sport**	**Number of Athletes 1yMSCL Number (% of total)**	**Number of Athletes 5yMSCL Number (% of total)**
**Soccer**	221 (12.4)	83 (12.1)
**Swimming**	153 (8.6)	62 (9.0)
**Ski/snowboard**	149 (8.4)	59 (8.6)
**Basketball**	141 (7.9)	58 (8.4)
**Football**	130 (7.3)	54 (7.8)
**Baseball**	145 (8.13)	54 (7.8)
**Dance**	122 (6.8)	38 (5.5)
**Track and Field**	86 (4.8)	31 (4.4)
**Cycling**	73 (4.1)	23 (3.3)
**Softball**	56 (3.1)	23 (3.3)
**Cheerleading**	45 (2.5)	22 (3.2)
**Golf**	66 (3.7)	22 (3.2)
**Tennis**	52 (2.9)	22 (3.2)
**Lacrosse**	40 (2.2)	21 (3.0)
**Hockey**	34 (1.9)	19 (2.8)
**Bowling**	60 (3.4)	18 (2.6)
**Volleyball**	65 (3.6)	17 (2.5)
**Wrestling**	30 (1.7)	17 (2.5)
**Other: Gymnastics, Cross Country, Karate, Field Hockey, Marching Band, Figure Skating, Weight lifting, Tae Kwon Do, Diving, various other sports/activities**	96 (5.4)	46 (6.7)
	Total: 1783	Total: 689

**Figure 1:**
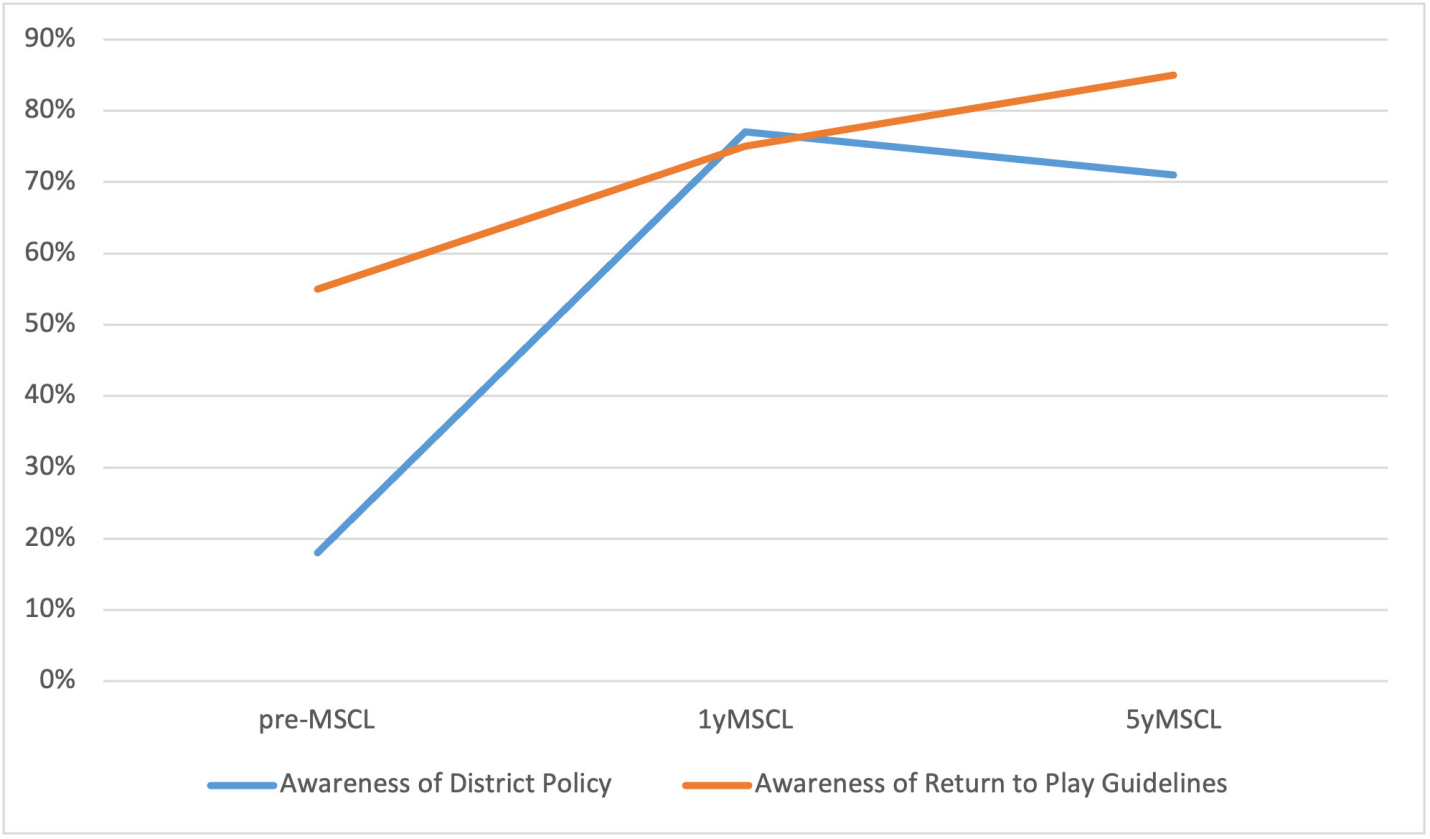
Changes in Parental Awareness of Sports Related Concussions

Approximately half (52%) of total athletes had been reportedly enrolled in a school-sponsored sport during the year prior to the survey. Awareness of a school district policy regarding concussions (Figure 1) was 71% (n=126) at 5yMSCL, down from 77% at 1yMSCL (p = 0.15), but still significantly higher than pre-MSCL levels. (18%, p < 0.0001)

Attitudes towards student health and sport participation remained largely unchanged across the three surveys. In both post-MSCL surveys, 100% of parents and guardians felt they were responsible for teaching their child to make healthy choices, essentially unchanged from the pre-MSCL (99.5%). Seventy-two percent (n=128) of respondents felt it was important for their child to succeed in his/her sporting activities, unchanged from 71% in the 1yMSCL (p = 0.88) and 68% at pre-MSCL. (p = 0.44)

One area of changing attitudes was noted with 116 (65%) of respondents at 5yMSCL feeling that playing competitive sports as a child would increase their professional success as an adult, down from 72% at 1yMSCL (p = 0.08) and 75% at pre-MSCL (p = 0.02). In summary, parent respondents did not feel sports were as important to their children’s’ future professional success with maturation of the MSCL.

Cultural perceptions regarding safety and head injuries in athletics also remained largely unchanged. At five years post-MSCL, only 93 (52%) of respondents felt enough attention was being paid to safety in organized sports, unchanged from 51% at 1yMSCL (p = 0.95). Pre-MSCL, 43% (p = 0.09) of respondents felt there was enough attention paid to safety.

Compared to professional sports, 149 (84%) of respondents felt amateur sports should observe modified rules for safety. Again, this was unchanged from the previous studies (84% at 1yMSCL and pre-MSCL; p = 0.70, p = 0.96). Notably, 46 (26%) of respondents felt that fighting in ice hockey was an acceptable tradition, which is unchanged from previous surveys (1yMSCL 28%, p = 0.68; pre-MSCL 30%, p = 0.35).

Most (n = 141, 79%) 5yMSCL respondents felt that they could recognize a concussion in their child, which had increased over the prior two surveys (74% in 1yMSCL, p = 0.17; 63% in pre-MSCL, p = 0.0003). After a child had sustained a head injury, 36 (20%) of respondents believed that they could determine whether their child was ready to return to play which was unchanged from 1yMSCL (20%, p = 0.93). However, this was increased from pre-MSCL levels, in which only 10% of respondents indicated that they could determine their child’s readiness to return to sports (p = 0.003).

**Figure 2:**
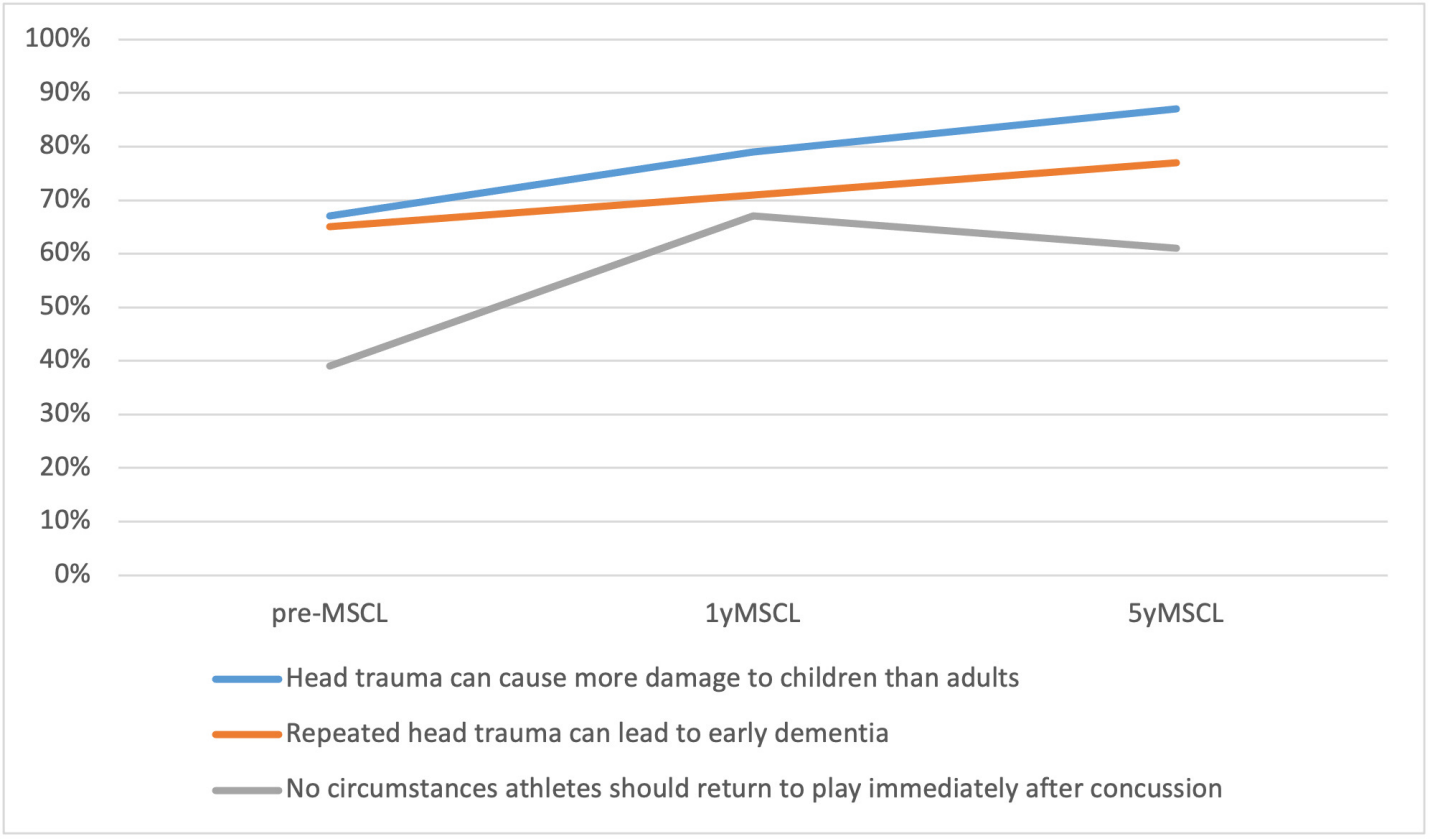
Changes in Parental Sports-Related Concussion Knowledge

Overall, parental knowledge on concussions had increased over the course of the three surveys (Figure 2) When asked if head trauma during childhood could cause more damage to their developing brains compared to adults, 155 (87%) respondents agreed, representing a significant increase from both the 1yMSCL survey (78%, p = 0.03) and pre-MSCL survey (67%, p < 0.0001).

One hundred thirty seven (77%) respondents also agreed that repeated head trauma in teen years could lead to early dementia, increasing from 71% (p = 0.13) at 1yMSCL and 65% (p = 0.01) at pre-MSCL. Most encouraging, 151 (85%) of parents reported that they were aware of medical guidelines determining when an athlete should return to play after SRC This increased from 75% (p = 0.01) at 1yMSCL and 55% (p < 0.0001) pre-MSCL. (Figure 1)

When asked if there were circumstances during which their athlete could return to play immediately after sustaining a concussion, 109 (61%) answered “No.” At 1yMSCL this was 67% (p = 0.20). This was still much higher than pre-MSCL where only 39% (p < 0.0001) responded that there were no circumstances allowing immediate return to play. Over a third of parents (34%) reported they didn’t know if there were circumstances that would allow immediate return to play.

Respondents in each of the three surveys felt that parents should know the signs and symptoms of concussion. All respondents (178 or 100%) also believed that coaches should receive special training regarding head injury management, largely unchanged from previous surveys (98% 1yMSCL, p=0.09; 100% pre-MSCL, p = 1.00). Nearly 100% of respondents (i.e., at least 98% or 174) for the three surveys felt their child should be evaluated by a doctor after sustaining a head injury before returning to play. Parents and guardians were also asked about their awareness of the Michigan law regarding SRC. Of all respondents, 73 (41%) indicated that they were aware of the MSCL which was consistent with previous survey responses (41%, p = 0.88).

**Figure 3:**
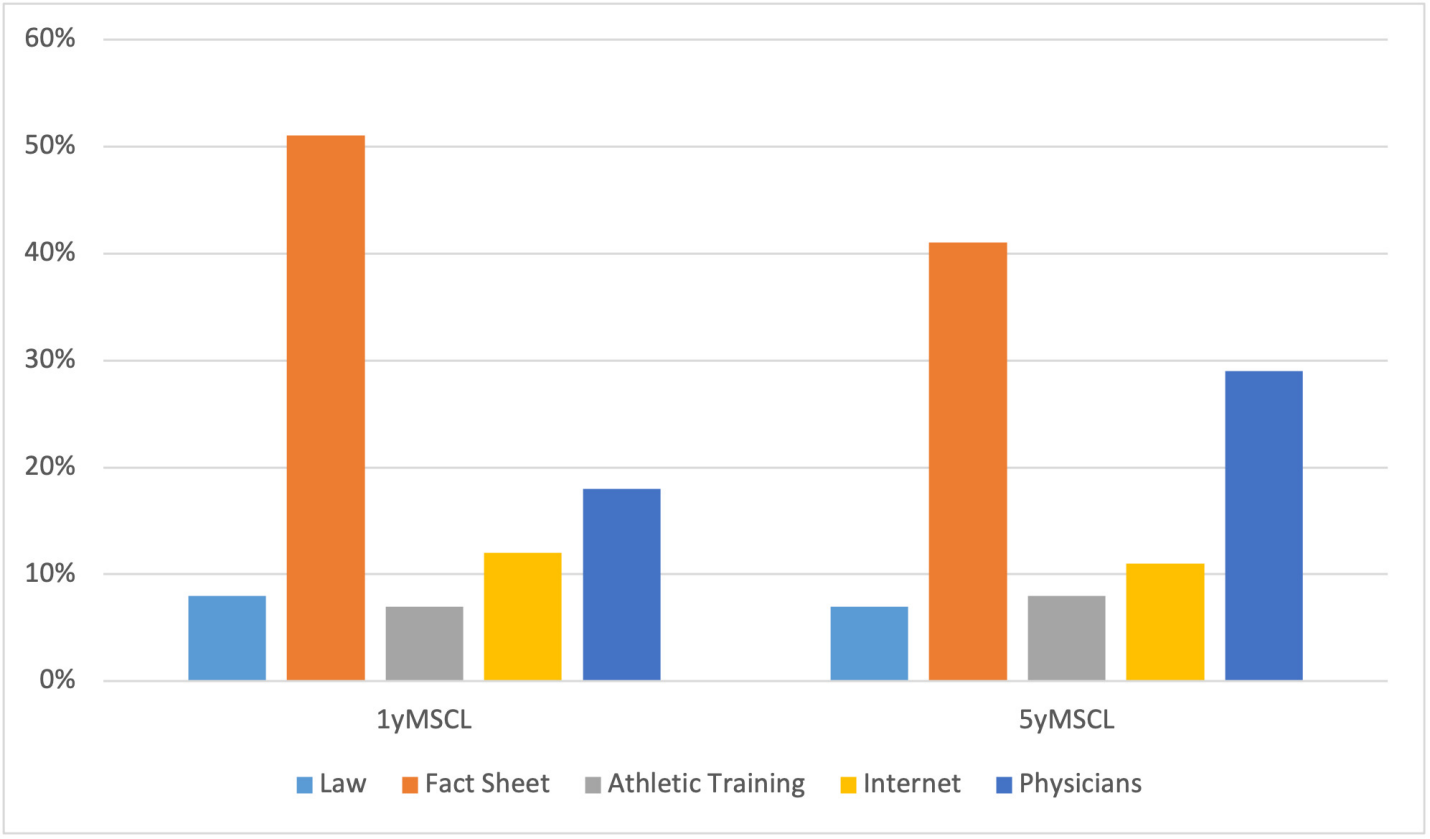
Sports-Related Concussion Resources ranked as “Most Helpful”

Finally, parents were asked to rank the resources they found most helpful regarding concussion education. (Figure 3) Notably, 75 (42%) of total respondents ranked the concussion fact sheet as most helpful, with 139 (78%) of those who recalled having received the handout ranked it as most helpful. Like the 1yMSCL, the next most helpful resource was physicians, with 52 (29%) of respondents ranking them as the most helpful educational resource. The least helpful resources for concussion safety information were the MSCL wording, sport-specific athletic training, and other information obtained from the internet.

One survey area that appeared to influence response differences across sample subgroups was whether parents felt they had the ability to recognize a concussion in their athlete. In those who recalled having received the concussion fact sheet handout, 18 (25%) answered “Strongly Agree” compared to 14 (13%) in the group who did not recall receiving the handout. Those who recalled the handout were also more likely (52 or 71%) to answer “No” to the existence of circumstances that might allow return to play immediately after sustaining a head injury. Those who did not recall receiving the handout were less likely (47 or 45%) to answer “No” to this question.

## DISCUSSION

According to the 2016 consensus statement of the Consensus in Sport Group (CISG), concussion can be broadly defined as “a traumatic brain injury induced by biomechanical forces” that is further characterized by a set of features relating to mechanism, neuropathological changes and clinical sign and symptoms. The CISG has continued to adapt and change this definition as expert opinions have evolved, reflective of ongoing research gaps concerning clinicians’ understanding of the pathophysiological factors behind concussion.^6^

Since 2007, all 50 states have passed some type of legislation addressing youth SRC. However, not all states currently require coaches or trainers to undergo formal training.^18^ Furthermore, although many states require that parents have access to educational material (e g., forms posted on a website or distributed), they do not require parental acknowledgment of receipt or informed consent prior to their youth athlete participating in sports.^19^

Given our findings, we have concluded that mandating parents to acknowledge receipt of educational materials may enhance their ability to recognize concussion in their student athletes and be more familiar with return-to-play practices.^20–22^

Of our sample respondents, 17% reported having personally sustained a prior head injury, compared to nearly one-third (31%) of their student-athletes. This finding could represent changes in diagnosing SRC across generations.^23–25^ This increase may reflect increasing athletic participation levels^26^ or represent better education and recognition of concussion by coaches, trainers, doctors and lay-persons.^27,28^

The largest sample subgroup of parents and guardians reported that the concussion educational handout they had received was the most helpful source of information. As not all states now require this type of parent education, we have concluded that further nationwide legislation would provide parents with the necessary tools to recognize concussion in their youth athletes and contribute to improved overall safety in athletics.

### Study Limitations

Our study results were largely obtained from middle-class suburban Michigan parents, with most respondents having completed higher education levels. A 2015 study showed that parental concussion knowledge correlated with education level.^29^ This may decrease the generalizability of our results to more educationally diverse settings. Selection and “preferred response” biases may have also skewed the responses we obtained.

## CONCLUSIONS

As parents’ SRC knowledge levels increase, multiple factors besides the implementation of legislation and refinement of educational materials may be responsible. Increased media attention concerning SRC and sizable NFL traumatic brain injury settlements (e.g., including the movie *Concussion*^30^) have likely influenced parental attention to this issue. These results suggest that parents and guardians of student athletes may derive increased SRC knowledge and awareness of medical guidelines from maturation of the MSCL and increased SRC mainstream media attention.

### Conflicts of Interest

The authors have none to report.

## APPENDIX

### Survey: Sports-Related Head Injuries in Student Athletes

1. Dear Participant,
  You are invited to participate in a research study being conducted by the faculty of the Genesys Health System. The purpose of the research is to determine what parents and guardians know about head injuries suffered by children and teens while playing sports. You are one of approximately 6500 people that are invited to participate in this project. Your participation in this research project is completely voluntary. You may decline altogether or leave blank any survey questions you don’t want to answer. There are no known risks to participation beyond those encountered in everyday life. Your responses will be confidential and data from this research will be reported only as a collective combined total. No one will know your individual answers to this questionnaire. We will send the results of this study via this email system when our project is complete. If you agree to participate in this project, please click on the link below to complete the survey. Answer the questions on the survey as best you can. It should take approximately 10-15 minutes to complete. Be sure to answer the last question, which is your vote for the school that will receive the 50 new bike helmets. The method of distribution will be determined by the school staff. Each helmet will be professionally fit by a safety specialist and the recipient will be instructed about its proper use. If you have any questions about this project, please feel free to contact Dr. Karyn Liebsch at karyn.liebsch@ascension.org. Information on the rights of human subjects in research is available through the Institutional Review Board, One Genesys Parkway, Suite 2442, Grand Blanc, MI 48439; phone (810) 606-7722. Thank you for your assistance in this important endeavor. Sincerely yours,
  Karyn Liebsch, DO
  Virginia LaBond, MS MD FACEP Do you agree to participate?
  - Yes
  - No
2. Are you the parent and/or guardian to a student enrolled in the Clarkston School District?
  - Yes
  - No
3. What is your relationship to the student/students?
  - Mother
  - Father
  - Other
4. If you answered "other" to question #3, please enter your relationship here.
5. Check all grades that your student/students currently attend:
  - Preschool/Kindergarten
  - Grades 1-5
  - Grades 6-7
  - Grades 8-9
  - Grades 10-12
6. Select all sports your student/students have been involved in:
  - Football, Baseball, Soccer, Basketball, Lacrosse, Tennis, Swimming, Diving, Cheer, Dance, Hockey, Track & Field, Wrestling, Volleyball, Softball, Golf, Bowling, Ski/Snowboarding, Equestrian, Cycling, Other
7. If you answered other on #6, please specify:
8. In the past year, have your students participated in a school-organized sport?
  - Yes
  - No
9. If yes, did you receive an informational handout about concussions?
  - Yes
  - No
10. Have any of your students ever sustained a head injury requiring medical attention?
  - Yes
  - No
11. Have you ever sustained a head injury requiring medical attention?
  - Yes
  - No
12. What is your age in years?
13. What is your gender?
  - Male
  - Female
  - Other
14. What is your highest level of education?
  - Some high school
  - High school graduate
  - Some college
  - Trade school
  - Bachelor’s degree
  - Graduate degree
15. Does Clarkston Community School District have a policy on sports related head injuries?
  - Yes
  - No
  - Don’t know
16. Are there some circumstances under which a student athlete can return to play immediately after sustaining a concussion?
  - Yes
  - No
  - Don’t know
17. Are there medical guidelines to determine when a student athlete should return to play after concussion?
  - Yes
  - No
  - Don’t know
18. Repeated head trauma in the teen years can result in permanent early dementia.
  - True
  - False
  - Don’t know
19. Head trauma can cause more damage in children than in adults because their brains are still developing.
  - Yes
  - No
  - Don’t know
20. There is enough attention paid to safety in organized sports.
  - Strongly Agree
  - Agree
  - Disagree
  - Strongly Disagree
21. After having a concussion and before returning to play, a child should be evaluated by a doctor.
  - Strongly Agree
  - Agree
  - Disagree
  - Strongly Disagree
22. Coaches should be required to have training on head injury management.
  - Strongly Agree
  - Agree
  - Disagree
  - Strongly Disagree
23. All parents should know the signs and symptoms of a concussion.
  - Strongly Agree
  - Agree
  - Disagree
  - Strongly Disagree
24. Compared to professional sports, amateur sports should have modified rules that ensure safety.
  - Strongly Agree
  - Agree
  - Disagree
  - Strongly Disagree
25. I can recognize a concussion in my child/student.
  - Strongly Agree
  - Agree
  - Disagree
  - Strongly Disagree
26. After a concussion, I can determine when my child/student is ready to return to playing sports.
  - Strongly Agree
  - Agree
  - Disagree
  - Strongly Disagree
27. Playing competitive sports as a child will increase an individual's professional success as an adult.
  - Strongly Agree
  - Agree
  - Disagree
  - Strongly Disagree
28. I am responsible for teaching my child/student to make healthy choices.
  - Strongly Agree
  - Agree
  - Disagree
  - Strongly Disagree
29. Fighting in professional hockey is an acceptable tradition.
  - Strongly Agree
  - Agree
  - Disagree
  - Strongly Disagree
30. It is important that my child/student succeed in his/her sporting activities.
  - Strongly Agree
  - Agree
  - Disagree
  - Strongly Disagree
31. Are you aware of the new Michigan Law (Public Acts 342 and 343 of 2012) regarding concussions and sports-related head injuries?
  - Yes
  - No
32. In your experience, which of the following sources have been the most helpful for learning about concussion safety. Please rank them 1-5, with 1 being the most helpful and 5 being the least helpful.
  - Michigan Law (Public Acts 342 and 343)
  - Concussion fact sheet and acknowledgement form that was signed and returned to the school
  - Changes in athletic training of your child's/children’s' sport (tackling techniques, practice durations)
  - Internet (Google searches, social media, etc.)
  - Physicians and health care professionals
33. Which Clarkston school would you like to receive 50 new bike helmets? The method of distribution will be determined by the school staff. Each helmet will be professionally fitted by a safety specialist and the recipient will be instructed in its proper use.
  - Andersonville Elementary
  - Bailey Lake Elementary
  - Clarkston Elementary
  - Independence Elementary
  - Pine Knob Elementary
  - North Sashabaw Elementary
  - Springfield Plains Elementary
  - Sashabaw Middle School
  - Clarkston Junior High School
  - Clarkston High School
  - Renaissance High School
  - Early Childhood Education
